# Effect of temperature fluctuation on the localized pattern of action potential in cardiac tissue

**DOI:** 10.1038/s41598-020-72188-z

**Published:** 2020-09-15

**Authors:** Clovis Ntahkie Takembo, Henri Paul Ekobena Fouda

**Affiliations:** 1 Department of Electrical and Electronic Engineering, College of Technology, University of Buéa, P.O. Box 63, Buéa, Cameroon; 2 Laboratory of Biophysics, Department of Physics, Faculty of Science, University of Yaoundé I, P.O. Box 812, Yaoundé, Cameroon

**Keywords:** Biophysics, Physics

## Abstract

Based on the improved FitzHugh–Nagumo myocardial model driven by a constant external current, the effect of temperature fluctuation in a network of electrically coupled myocardial cells are investigated through analytical and numerical computations. Through the technique of multiple scale expansion, we successfully reduced the complex nonlinear system of equations to a more tractable and solvable nonlinear amplitude equation on which the analysis of linear stability is performed. Interestingly from this analysis, a plot of critical amplitude of action potential versus wave number revealed the growth rate of modulational instability (MI) is an increasing function of the thermoelectric couplings; $T^{(1)}$ and $T^{(2)}$, under fixed conditions of nonlinear electrical couplings. In order to verify our analytical predictions through the study the long-time evolution of the modulated cardiac impulses, numerical computation is finally carried out. Numerical experiment revealed the existence of localized coherent structures with some recognized features of synchronization. Through the mechanism of MI, changes in thermoelectrical couplings promote wave localization and mode transition in electrical activities in the cell lattice. Results could provide new insights in understanding the underlying mechanism of the manifestation of sudden heart disorder subjected to heavily temperature fluctuation.

## Introduction

Understanding mode transition in electrical activity in cardiac tissue from a normal rhythm to various pathological states has aroused the interest of several researchers^1–4^. The electrical activity in cardiac tissue and excitable network have been shown to be affected by a number of factors. These include internal factors like distribution of ion channel, topological set up, ionic permeability and intrinsic noise. External factors such as external stimulus current, environmental noise and temperature. Systemic temperature may remain constant during various cardiac physiological processes but in the event of clinical surgery and many other environmental fluctuation, temperature variation affects cardiac electrical activities. In fact, temperature variation influences excitability by regulating the conductance and gating kinetics of ion channel^5,6^. Xu et al.^5^, using memristive ion channels embedded on cell membrane reported the dependence of ion channels conductance on temperature fluctuation. The frequency and amplitude of action potential were observed to be modulated by temperature change. Indeed, thermoelectric coupling has been reputed for modifying complex spatiotemporal pattern of excitable media^7^. Benndorf et al.^8^ reported the opening time of ion channel increases greatly with a decreased in temperature. Readers can find many other reports on the effect and possible biological implications of intrinsic and environmental temperature variation in excitable media in the following references^9,10^. Temperature affects the time constant of the local kinetic reactions thereby inducing a degree of heterogeneity in the tissue^11^. Many modeling and computationally-oriented investigations including several experimental studies have confirmed substantial differences in conduction speed and spiral drift of chaotic electric potential propagation due to temperature fluctuation^12^.

In addition, the heart functioning relies on the collective dynamics of myocardial cells communicating through voltage-senstive gap junction proteins^13^. The nature of the coupling function among individual dynamical units affects significantly this collective dynamics. Complex spatiotemporal patterns attributed to the coupling topologies have been reported in several excitable networks^15–17^. For example, chimera states have been observed in non locally, globally, locally or nearest neighbor coupled networks and even complex networks^17^. Inhomogeneity within the cardiac tissue supports the existence of nonlinearity in the coupling function which plays an important role in modulating intercellular communication among the dynamical units.

In this paper, a realistic model for cardiac electrical activity is proposed from the standard two variables FitzHugh–Nagumo equations, where the effect of temperature change and nonlinear coupling are included. These factors influence on the network dynamics in relation to pattern formation and synchronization have been promptly studied through the technique of modulational instability (MI). Indeed, MI is a well known and documented mechanism in nonlinear physics. This ranges from fluid dynamics^22^, Bose Einstein condensate^23^, neurons electrical activities^24^ and cardiac action potential dynamics^25^. MI leads to the formation of soliton and wave trains in dynamical system due to the interactive effects of dispersion and nonlinearity. With complex spatiotemporal cardiac dynamics widely observed experimentally as a result of fast pacing and their connection with nonlinear diffusion, there has been growing need for a realistic model capable of discerning the mechanistic description of this pattern. Using the improved model proposed in this work, we show numerically the present of discrete localized wave patterns under the effects of nonlinear and thermoelectric couplings. The numerical experiment agrees with our analytical predictions.

## Model description

The qualitative local dynamical behaviors in neuronal electrical activities notably regular bursting, chaotic bursting, rhythmic spiking can be sufficiently captured by the FitzHugh–Nagumo (FHN)^18,19^ models. Aliev et al. modified the FHN model so that it describes adequately the dynamics of pulse propagation in the canine myocardium^20^. Indeed, the revised version of the FHN are often used to describe cardiac tissue electrical activities^4,21,25^. The evolution of the network dynamics under temperature fluctuation and nonlinear coupling^26^ in cardiac tissue is described by:

1 $$\begin{aligned} \begin{aligned} \frac{dv_{n}}{dt} &=\frac{f(v_{n},w_{n})}{T^{(1)}} +D(v_{n})(v_{n+1} -2v_{n}+v_{n-1}) + I_{0} ,\\ \frac{dw_{n}}{dt} &=\frac{g(v_{n},w_{n})}{T^{(2)}}, \end{aligned} \end{aligned}$$

where $n=1,2,\ldots ,N$ and *N* represents the position of excitable node in the network. The variables $v_{n}$ and $w_{n}$ describe the activator and the inhibitor of the $n^{th}$ node. $I_{0}$ is the stimulation current, $T^{(1)}$ and $T^{(2)}$ are the thermoelectric couplings. The nonlinear reaction functions $f(v_{n},w_{n})$ and $g(v_{n},w_{n})$ identify a discrete FHN model, which are capable of reproduces generic dispersion and restitution characteristics of cardiac tissue. They are given by:

2 $$\begin{aligned} \begin{aligned} f(v_{n},w_{n})&=- kv_{n}( v_{n} - a)(v_{n} - 1.0) - v_{n}w_{n}\\ g(v_{n},w_{n})&=\left( \varepsilon + \frac{\mu _{1}w_{n}}{v_{n} + \mu _{2}}\right) [ - w_{n} - kv_{n}(v_{n} - a_{0})]. \end{aligned} \end{aligned}$$

For simplicity and to be consistent with previous works, we set the system parameters at $I_{0}=1.2, k=8.0, a_{0}=0.15, \mu _{1}=0.2, \mu _{2}=0.3, \varepsilon =0.002.$ Cardiac tissue are characterized by multiple physical couplings, whereby myocardial cells communicate through voltage-sensitive gap junction proteins^13^. The voltage-dependent gap junction coupling for synaptic connection is given by the nonlinear function $D(v_{n})$:

3 $$\begin{aligned} \begin{aligned} D(v_{n})=D_{0}+D_{1}v_{n}+D_{2}v_{n}^{2}. \end{aligned} \end{aligned}$$

Equation (3) accounts for electrical couplings, with $D_{0}$ as the standard nearest-neighboring interaction strength of the gap junction, enriched by linear $D_{1}$ and quadratic $D_{2}$, couplings. To be consistent with previous work on non-linear diffusion in cardiac electrophysiology^14,26^, we set $D_{0}=0.02, D_{1}=0.50,$ and $D_{2}=0.85$. The nonlinear inhibitor or slow variable ion current term is expanded. The realistic system of Eq. (1) reduces to:

4 $$\begin{aligned} \begin{aligned} T^{(1)}\frac{dv_{n}}{dt} + \alpha _{0}v_{n}&=- kv_{n}^3 + \alpha _{1}v_{n}^2 - v_{n}w_{n}] + I_{ext}+ D_{0}T^{(1)}(v_{n+1} -2v_{n}+v_{n-1}) \\&\quad + D_{1}T^{(1)}v_{n}(v_{n+1} -2v_{n}+v_{n-1})+D_{2}T^{(1)}v_{n}^{2}(v_{n+1} -2v_{n}+v_{n-1}),\\ T^{(2)}\frac{dw_{n}}{dt} + \varepsilon w_{n}&= \beta _{0}v_{n} + \beta _{1}w_{n}^2 + \beta _{2}v_{n}^2 + \beta _{3}v_{n}w_{n} + \beta _{4}v_{n}^2w_{n} + \beta _{5}v_{n}w_{n}^2, \end{aligned} \end{aligned}$$

with $$\begin{array}{ccc} \alpha _{0}=ak &{} \beta _{0}=a_{0}\varepsilon k &{} \beta _{3}=ka_{0}\frac{\mu _{1}}{\mu _{2}} \\ \alpha _{1}=ka_{0} &{}\beta _{1}=-\frac{\mu _{1}}{\mu _{2}} &{}\beta _{4}=k\beta _{1}(1+\frac{a_{0}}{\mu _{2}}) \\ \beta _{2}=-\varepsilon k &{} \beta _{5}=\frac{\mu _{1}}{\mu _{2}^{2}}&{} I_{ext}=I_{0}T^{(1)}. \end{array}$$

The system of equations above are then reduced to a more tractable nonlinear amplitude equation using the discrete multiple scale expansion proposed by Leon et al.^27,28^.

## Method

Equation (4) obtained above is a system of nonlinear differential equations with no exact analytical solution. There exist several techniques of converting these equations into more integrable form^25,29,30^. Here, we make use of the reductive perturbation method popularly known as the multiple scale expansion method. The discrete multiple scale expansion is an interesting technique developed by Leon and Manna^27,28^. It’s an asymptotic analysis of a perturbation series, based on the existence of different scales, with the amplitude and the carrier wave both kept discrete. This expansion enables the deduction of a more manipulable equation from the model without losing their vital characteristics features. It has recently being widely extended to many other physical and biological media^4,31,32^.

Through the multiple scale analysis expansions, the first particle of chain network $(n=0)$, is set into oscillations when subjected to an external forcing with the natural frequency $\Omega$. As a result of nonlinearity present in the media, which naturally exists and affects real systems, the natural frequency deviates, with actual frequency $\omega$ and wave number *q*, becoming $\omega =\Omega _{0}+\epsilon \lambda$ and $q=q_{0}+\epsilon \frac{\lambda }{V_{g}}+\epsilon ^2C_{g}\lambda ^2+\cdots ,$ with $\frac{1}{V_{g}}=(\frac{\partial q}{\partial \omega })_\Omega$ being the group velocity and the group velocity dispersion $2C_{g}=(\frac{\partial ^2q}{\partial ^2\omega })_\Omega$. Where $\lambda$ is a small deviation from the natural frequency $\Omega _{0}$. If $\epsilon =0$, the frequency $\omega$ reduces to the natural frequency $\Omega _{0}$ of the system. The state vector $U_{n}(t)=\{{ v_{n}(t), w_{n}(t)}\}$ are used to summarize the main variables of Eq. (4). The generalized form of the unperturbed expressions are taken in the form

5 $$\begin{aligned} \begin{aligned} v_{n}(t)&=\int d\omega {\hat{\theta }}(\omega )e^{i(\omega t+q_{0}n)},\\ w_{n}(t)&=\int d\omega {\hat{\chi }}(\omega )e^{i(\omega t+q_{0}n)}. \end{aligned} \end{aligned}$$

with ${\hat{U}}(\omega )=\{{{\hat{v}}(\omega ),{\hat{w}}(\omega )}\}$. Where the expanded $\omega$ and *q* along with change of variables $\tau _{n}=\epsilon (t+n/V_{g})$ and $\zeta _{n}=\epsilon ^{2}n$ and with the condition $C_{g}=1$, the generalized trial solutions take the form

6 $$\begin{aligned} \begin{aligned} v_{n}(t)&=A(n,t)\theta (\zeta _{n},\tau _{n}),\\ w_{n}(t)&=A(n,t)\chi (\zeta _{n},\tau _{n}), \end{aligned} \end{aligned}$$

where $A(n,t)=e^{i(qn+\omega t)}$. A new lattice number *m* is introduced to support a large grid. Thus for a given lattice number *n*, only the set of lattice points $\ldots ,n-N, n, n+N,\ldots$ can be indexed in terms of the slow variable *m* as $\{\ldots ,(n-N)\rightarrow (m-1)$, $n\rightarrow m$, $(n+N)\rightarrow (m+1)\ldots ,\}$, where $N=1/\epsilon ^2$ is assumed. We recall that since the model under studies considers discreteness to be highly pronounced, this justifies the above assumption for *N*^31^. In so doing, the slow modulation $U(\zeta _{n},\tau _{n})$ of the plane wave *A*(*n*, *t*) can be replaced by the functions $U(m,\tau )$, with $\tau =\tau _{n}$, and making use of the Fourier series in power of the parameter $\epsilon$

7 $$\begin{aligned} \begin{aligned} v_{n}(t)&=\sum _{p=1}^{\infty }\epsilon ^{p}\sum _{l=-p}^{p}\theta _{p}^{l}(\zeta _{n},\tau _{n})A^{l}(n,t),\\ w_{n}(t)&=\sum _{p=1}^{\infty }\epsilon ^{p}\sum _{l=-p}^{p}\chi _{p}^{l}(\zeta _{n},\tau _{n})A^{l}(n,t), \end{aligned} \end{aligned}$$

With $\theta _{p}^{-l}(m,\tau )=(\theta _{p}^{l}(m,\tau ))^{*}$, and $\chi _{p}^{-l}(m,\tau )=(\chi _{p}^{l}(m,\tau ))^{*}$. The above solutions are inserted into Eq. (4) leading to a set of coupled equations. They can be solved at different orders of the small parameter $\epsilon$, with the corresponding harmonics *l*. As such, the leading order (1, *l*) yields a homogeneous set of equations where when $l=0$, we obtain:

8 $$\begin{aligned} \theta _{1}^{0}(m,\tau )=\chi _{1}^{0}(m,\tau )=0. \end{aligned}$$

Similarly, taking $l=1$ leads to a linear system whose determinant is null, yielding the dispersion relation:

9 $$\begin{aligned} \Big [i\omega T^{(1)}+ 4D_{0}T^{(1)}\sin ^{2}\Big (\frac{q}{2}\Big )+ \alpha _{0}\Big ]\times (i\omega T^{(2)} + \varepsilon )=0. \end{aligned}$$

According to the above dispersion relation, the non-trivial solutions $\theta _{1}^{1}(m,\tau )$ and $\chi _{1}^{1}(m,\tau )$ of the resulting homogeneous set of equations could be looked out in the form:

10 $$\begin{aligned} \begin{aligned} \theta _{1}^{1}(m,\tau )&=\psi (m,\tau ),\\ \chi _{1}^{1}(m,\tau )&=\frac{\beta _{0}}{\varepsilon + i\omega T^{(2)}}\times \psi (m,\tau ). \end{aligned} \end{aligned}$$

At the order (2, *l*), when $l=0$, the solutions of the resulting system equations give

11 $$\begin{aligned} \begin{aligned} \theta _{2}^{0}(m,\tau )=&F_{3}\times \mid \psi (m,\tau )\mid ^{2},\\ \chi _{2}^{0}(m,\tau )=&F_{0}\times \mid \psi (m,\tau )\mid ^{2}, \end{aligned} \end{aligned}$$

where,

12 $$\begin{aligned} \begin{aligned} F_{3}=&\left( \frac{2\alpha _{1}}{\alpha _{0}} - \frac{2\varepsilon }{\beta _{0}\alpha _{0}}\right), \\ F_{0}=&\left[ \frac{\beta _{0}}{\varepsilon }F_{3}+ \frac{2\beta _{1}}{\varepsilon \beta _{0}^{2}}(\varepsilon ^{2} - (i\omega T^{(2)})^{2}) +\frac{2\beta _{2}}{\varepsilon }\right] . \end{aligned} \end{aligned}$$

With a zero determinant, the case $l=1$ should satisfy the Fredholm condition

13 $$\begin{aligned} V_{g}= 2iD_{0}\sin (q). \end{aligned}$$

For the solution to be found in the form

14 $$\begin{aligned} \begin{aligned} \theta _{2}^{1}(m,\tau )&=\delta (m,\tau ),\\ \chi _{2}^{1}(m,\tau )&=\frac{\beta _{0}}{(\varepsilon + i\omega T^{(2)})}\delta (m,\tau ) - \frac{T^{(1)}}{\beta _{0}}\frac{\partial \psi (m,\tau )}{\partial \tau } \end{aligned} \end{aligned}$$

where $\delta (m,\tau )$ is taken to be an arbitrary function. At the same order, but for $l=2$, solutions are derived in the form

15 $$\begin{aligned} \begin{aligned} \theta _{2}^{2}(m,\tau )&=F_{1}\times \psi (m,\tau )^{2},\\ \chi _{2}^{2}(m,\tau )&=F_{2}\times \psi (m,\tau )^{2}, \end{aligned} \end{aligned}$$

where

16 $$\begin{aligned} \begin{aligned} F_{1}=&\frac{\beta _{0}(\alpha _{1}+4D_{0}\sin ^{2}\left( \frac{q}{2}\right) -(\varepsilon + i\omega T^{(2)})}{\beta _{0}(2i\omega T^{(1)}+4D_{1}\sin ^{2}(q) + \alpha _{0})}\\ F_{2}=&\frac{1}{(\varepsilon + 2i\omega T^{(2)})}\left[ \beta _{0}F_{1}+ \frac{\beta _{1}\beta _{0}^{2}}{(\varepsilon + i\omega T^{(2)})^{2}} + \beta _{2}\right] . \end{aligned} \end{aligned}$$

Finally, we solve the system for $\theta _{3}^{1}(m,\tau )$ and $\chi _{3}^{1}(m,\tau )$, that is for $p=3$ and $l=1$, which yields, while making use of the previous solutions, the amplitude equation in $\psi (m,\tau )=\psi _{m}$ as:

17 $$\begin{aligned} i\frac{P}{2}(\psi _{m+1}-\psi _{m-1})+Q\frac{\partial ^2\psi _{m}}{\partial \tau ^2}+R|\psi _{m}|^2\psi _{m}=0, \end{aligned}$$

with

18 $$\begin{aligned} \begin{aligned} P&=2D_{0}\sin (q),\\ Q&=\frac{D_{0}\cos (q)}{V_{g}^{2}},\\ R&=-3k+F_{0}+\left( 2\alpha _{1}+1-4D_{1}\sin ^{2}\left( \frac{q}{2}\right) \right) F_{1}+F_{2}\\ {}&+ \left( \frac{\beta _{0}}{\varepsilon + i\omega T^{(2)}}+2\alpha _{1}-4D_{1}\sin ^{2}\left( \frac{q}{2}\right) \right) F_{3}-4D_{2}\sin ^{2}\left( \frac{q}{2}\right) . \end{aligned} \end{aligned}$$

## Modulaional instability analysis

### Linear stability analysis

In resolving boundary value problems in optical fibres, the continuous version of Eq. (17) is a well-known model usually encountered^33^. Recently, this model has also been reported in biological media^24^. We build an approximate solution of Eq. (17) in the form

19 $$\begin{aligned} v_{n}(t) =\epsilon \psi (m,\tau )e^{i(qn - \omega t)} + \lambda (\epsilon ^{2}) + c.c, \end{aligned}$$

where $\psi (m,\tau )$ admits the stationary solution

20 $$\begin{aligned} \psi (m,\tau )=\psi _{0}e^{i(\vartheta m - \Gamma \tau )} \end{aligned}$$

with the wave number $\vartheta$ and the frequency of perturbation $\Gamma$. By inserting Eq. (20) in Eq. (17) we obtain the nonlinear dispersion relation given by

21 $$\begin{aligned} \Gamma ^2=\gamma (q)\times [|\psi _{0}|^2 -\frac{D_{0}\sin (q)}{R}\sin (\vartheta )]. \end{aligned}$$

It is expected that when $\Gamma ^2<0$, we should obtain unstable waves within the media. By considering the sign of $\gamma (q)=\frac{R}{Q}$ and $|\psi _{0}|^2 -\frac{D_{0}\sin (q)}{R}\sin (\vartheta )$ and taking into consideration that we are deally with a boundary condition problem then as $\vartheta$ is bounded due to discreteness effect, we set $\sin (\vartheta )=1$. The quantity $\gamma (q)<0$, implies MI result when $|\psi _{0}|^2 -\frac{D_{0}\sin (q)}{R}>0$. Thus

22 $$\begin{aligned} |\psi _{0}|^2>\frac{D_{0}\sin (q)}{R}=\psi _{0,cr}^{2}. \end{aligned}$$

When the above condition is not respected, the wave remains stable and will propagates via the lattice without distortion. The instability condition for $\gamma (q)<0$ can now be stated exclusively as

23 $$\begin{aligned} |\psi _{0}|^2>\psi _{0,cr}^{2}. \end{aligned}$$

It’s expected from the above analyzes that when the parameters fall inside the unstable region, unstable patterns of waves will be observed in the cell lattice through the activation of MI.

**Figure 1:**
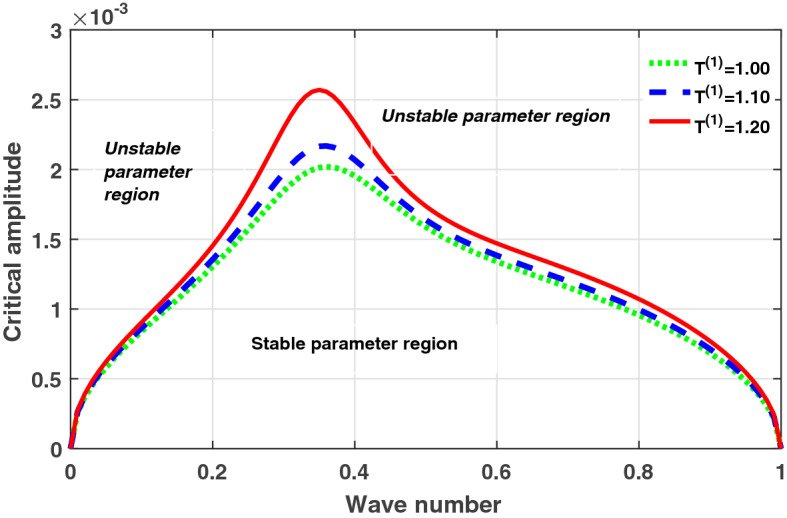
Plot of critical amplitude ($\psi _{0,cr}$) versus wave number (*q*) for different values of thermal coupling $T^{(1)}$. The rest of the system parameters set at $T^{(2)}=1.0$, $D_{0}=0.02, D_{1}=0.50,$ and $D_{2}=0.85$.

**Figure 2:**
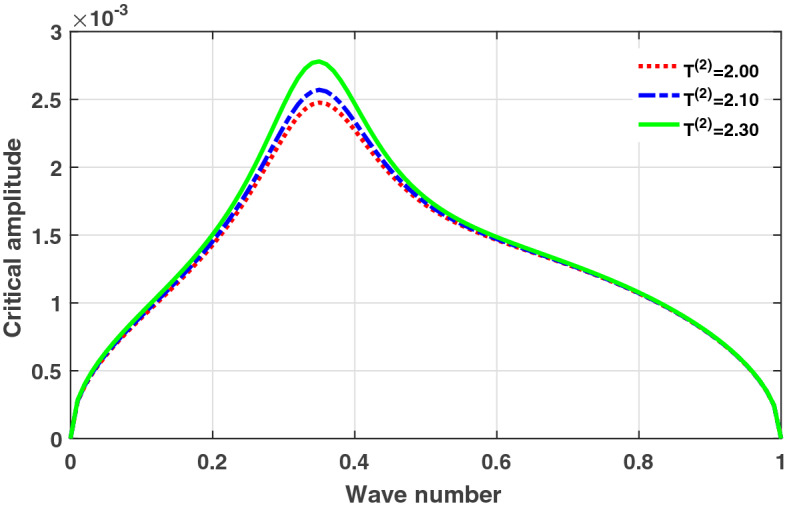
Plot of critical amplitude ($\psi _{0,cr}$) versus wave number (*q*) for different values of thermal coupling $T^{(2)}$. The rest of the system parameters set at $T^{(1)}=1.0$, $D_{0}=0.02, D_{1}=0.50,$ and $D_{2}=0.85$.

The analytical plots of the critical amplitude versus the wave number according to Eq. (23) for different thermoelectric couplings are depicted in Figs. 1 and 2. The stable/unstable regions of modulational instability (MI) are clearly portrayed in Fig. 1. The critical amplitude is observed to be an increasing function of the thermoelectric couplings $T^{(1)}$ and $T^{(2)}$. These results indicate a non negligible role of temperature fluctuation on the collective electrical activities of myocardial cells, through the promotion of MI. Based on the theory of MI, it is expected that when parameters of the system are picked from unstable regions, localized wave patterns will emerged in the network lattice, due to the concomitant effect of dispersion from the nonlinear electrical couplings and nonlinearity from the thermoelectrical couplings. A possible mechanism could that temperature fluctuation in the media induces a thermoelectric voltage which further modulates the electrical activities of cardiac tissue. This modulation consequently modifies the nonlinearity of the media which undoubtedly promotes the mechanism of MI, as revealed from the analytical plots. Numerical experiments are further performed in the next subsection to confirm our analytical results.

### Spatiotemporal patterns under thermoelectric couplings

**Figure 3:**
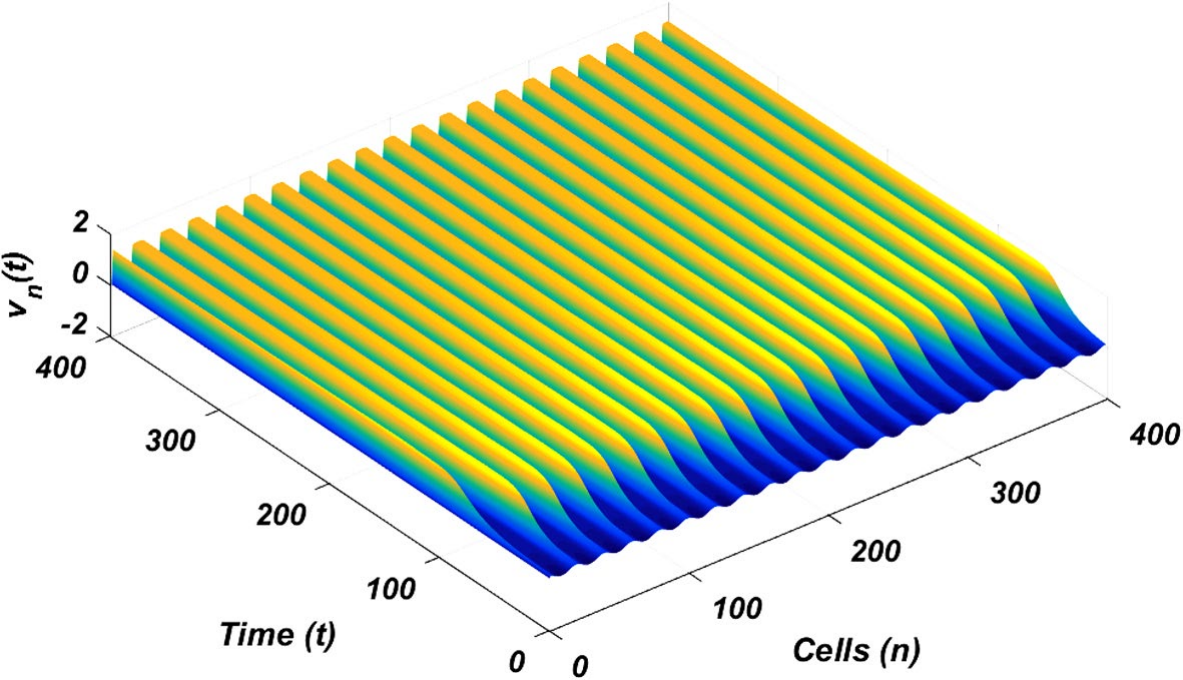
Panel shows 3D evolution pattern of the cardiac action potential for an array of 400 cells, for $T^{(1)}=1.4, T^{(2)}=2.4$, $D_{0}=0.02, D_{1}=0.50,$ and $D_{2}=0.85$.

**Figure 4:**
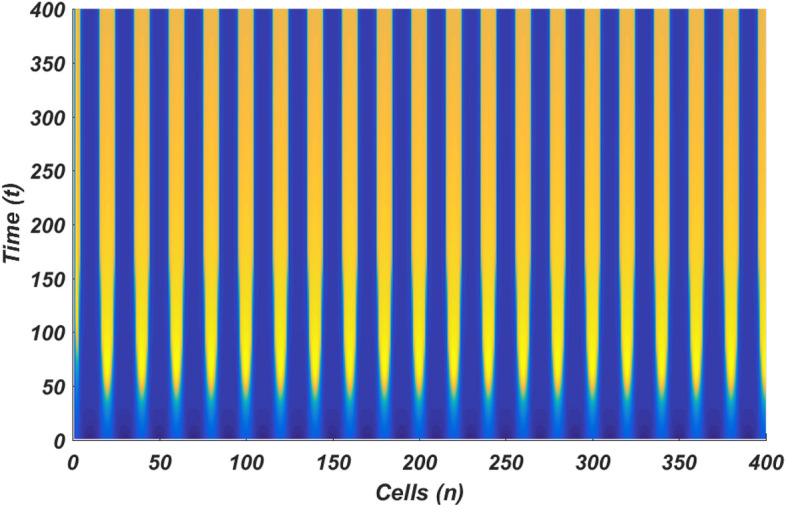
Spatiotemporal evolution (X,Y) of the cardiac action potential for an array of 400 cells presented in Fig. 3, for $T^{(1)}=1.4$ and $T^{(2)}=2.4$. Time increases from top to bottom.

**Figure 5:**
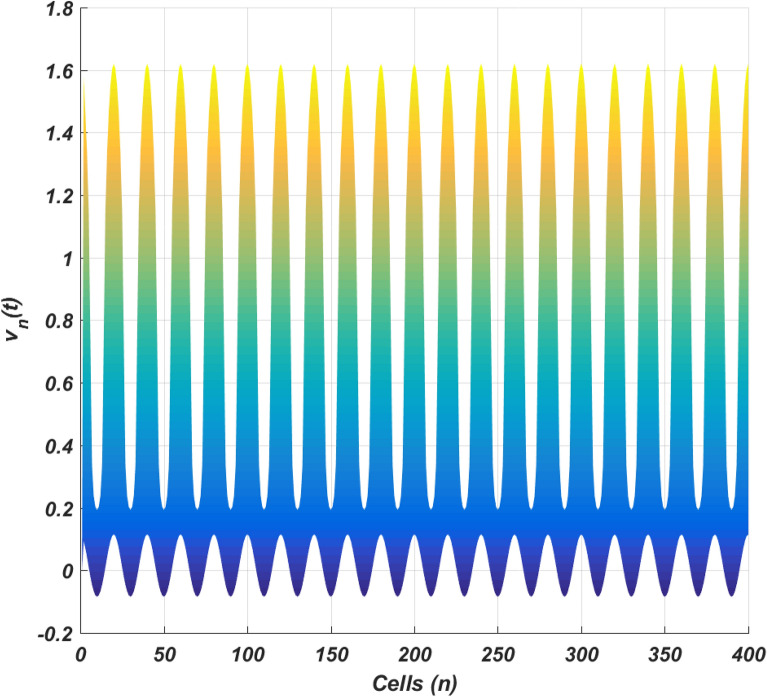
Spatial feature (Y, Z) of the 3D pattern presented in Fig. 3, for $T^{(1)}=1.4$ and $T^{(2)}=2.4$.

The linear stability analysis of MI discussed in the preceding subsection gives reasonable prediction of the non negligible role of thermoelectric couplings on the collective electrical activities amongst myocardial cells. However, the linear stability analysis does not give any insight into the long-time dynamics of the modulated plane waves under investigation. Thus in other to examine the long time evolution of the modulated wave in the network as well as to verify the analytical predictions, we carried out the numerical simulation of the Eq. (1) via fourth-order Runge Kulta Computational scheme. The initial conditions are carefully selected from the unstable region of MI, to correspond to plane waves slightly modulated. The wave numbers set at $q=0.21\pi$ and $\vartheta =0.10\pi$. By setting the thermoelectric couplings at $T^{(1)}=1.4$ and $T^{(2)}=2.4$, we present the patterns of electrical activities of cardiac tissue in Figs. 3, 4 and 5 for an array of 400 cells.

**Figure 6:**
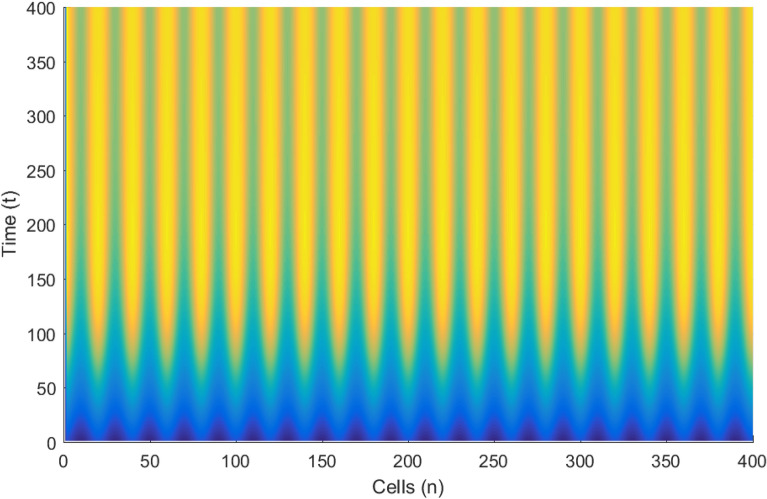
Spatiotemporal evolution pattern (X,Y) of 400 cells, for $T^{(1)}=6.0$ and $T^{(2)}=6.0$. The rest of the system parameters are kept fixed as in Fig. 3.

**Figure 7:**
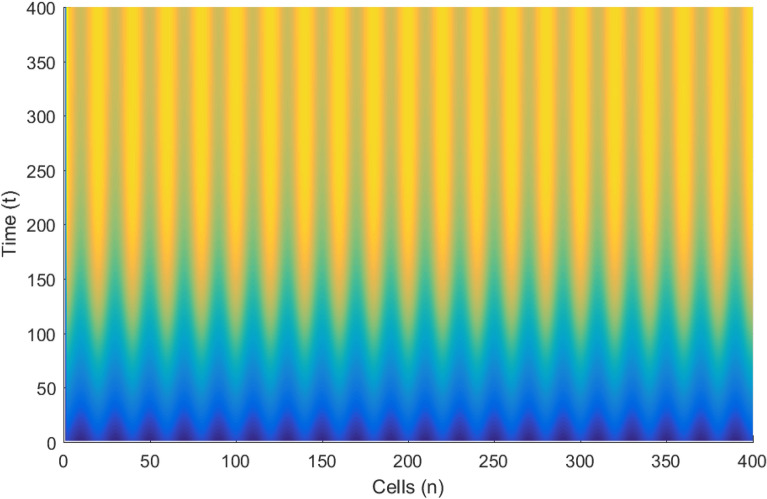
Spatiotemporal evolution pattern (X,Y) of 400 cells, for $T^{(1)}=8.0$ and $T^{(2)}=8.0$. The rest of the system parameters are kept fixed as in Fig. 3.

Figures 3, 4 and 5 portrait the features of the cardiac action potential of the network. Figure 3 presents the 3D patterns, Fig. 4 presents the spatiotemporal evolution and Fig. 5 presents the spatial features. They are clearly localized wave pattern. Firstly, the wave localization pattern observed, confirms our analytical predictions, based on the well documented theory of modulational instability, which predicts the existence of localized modes in all systems where dispersion and nonlinearity are present^22^. The modulated wave structures obtained though regular patterns are nonlinear waves since the thermoelectric couplings parameters ($T^{(1)}=1.4, T^{(2)}=2.4$) are selected from the unstable region of MI, as provided by Fig. 1. The bright regions observed in the spatiotemporal pattern as presented in Fig. 4 indicates where the neurons fire, while the blue regions quiescent state. These bright regions correspond to the firing mode of individual spikes inside a burst. The result obtained in this work also agrees with experimental observations from cultured cardiac myocyte^34,35^. Spatiotemporal dynamical patterns including the wave pattern obtained in this work in the form of discrete pulses have been reported in excitable network, physical and biological media^24,32,36,38^. Spatiotemporal patterns have been reported in excitable brain micro-circuits, described to be highly relevant in intercellular communication such as visual and olfactory cortices^37^. According to the analytical predictions, changing the thermoelectrical couplings should influence the patterns obtained. Figures 6 and 7 present the spatiotemporal evolution of the transmembrane potential for different values of $T^{(1)}$ and $T^{(2)}$.

**Figure 8:**
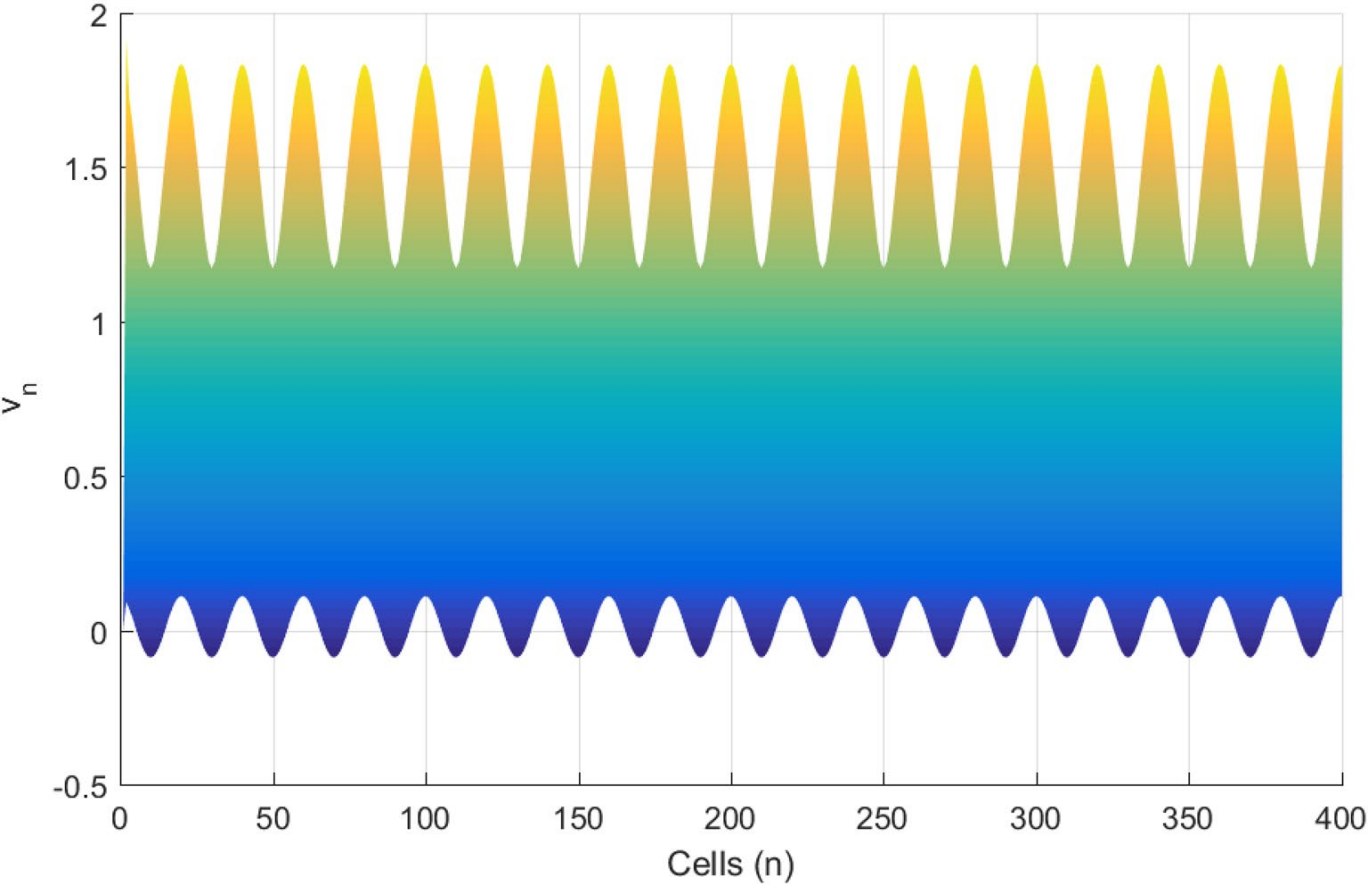
Spatial evolution (Y, Z) at $T^{(1)}=6.0$, $T^{(2)}=6.0$ of modulated wave for an array of 400 cells.

**Figure 9:**
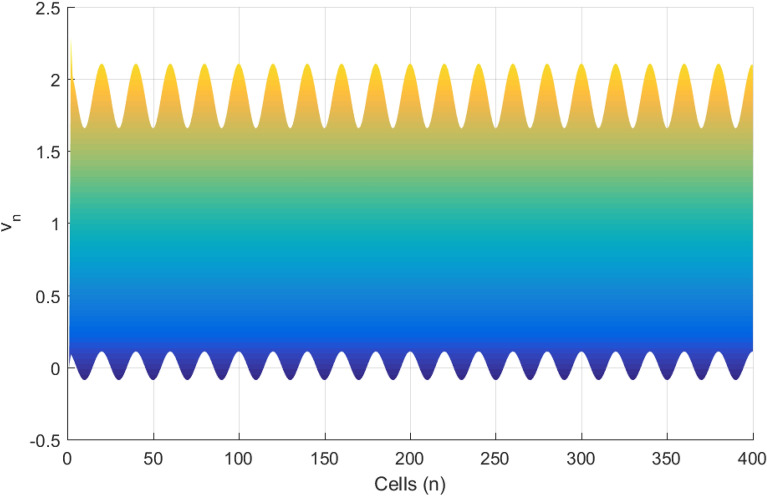
Spatial evolution (Y, Z) at $T^{(1)}=8.0$, $T^{(2)}=8.0$ of transmembrane potential for an array of 400 cells. The amplitude of modulated wave is observed to be an increasing function of the thermoelectrical couplings.

Figures 6 and 7 presents the spatiotemporal evolution pattern of the modulated wave, when the thermoelectrical couplings are increased, while keeping the rest of the system parameters constant. As expected from analytical predictions, the patterns emerging from the lattice are modified. As $T^{(1)}$ and $T^{(2)}$ are both changed from 6.0 (Fig. 6) to 8.0 (Fig. 7) respectively, we observe a delay in wave formation and localization in the lattice. The localized structure evolves towards a homogenous state, destroying discreteness. It could be thought that as the temperature of the media is increased, the resulting feedback modulation in the collective electrical activities increases the threshold transmembrane potential required for the emergence and propagation of an action potential. The disappearance of the discrete wave pattern suggests the possible assimilation of the network dynamics into a single myocardial cell behavior. In order to quantitatively characterize the changes in the amplitude of the modulated wave, we present the corresponding spatial patterns (presented in Figs. 6 and 7) in Figs. 8 and 9.

**Figure 10:**
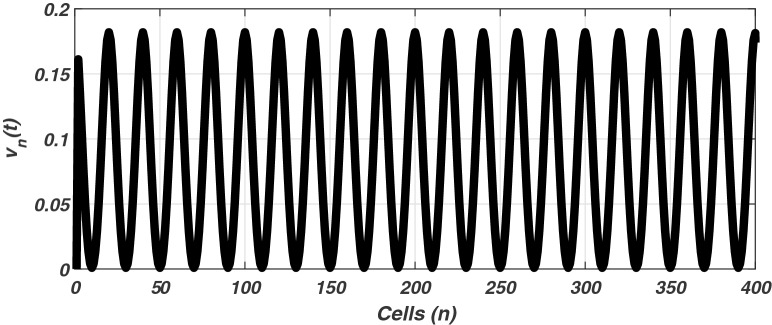
Spatial features (Y, Z) with $T^{(1)}=2.0$, $T^{(2)}$=2.0 at the time unit $t=50$.

**Figure 11:**
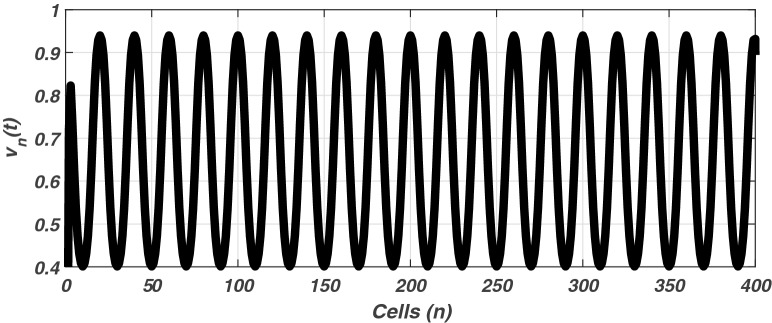
Spatial features (Y, Z) with $T^{(1)}=2.0$, $T^{(2)}$=2.0 at the time unit $t=250$.

Figures 8 and 9 show a rise in the modulated wave amplitude as the value of thermoelectrical couplings are increased. Such growth will surely promote the break down of the modulated wave propagating in the network into trains of solitonic objects. From biophysical point of view, a great variation in temperature from both the internal and external environment could initiates mode transition in the electrical activities of cardiac tissue, while a low value, the myocardial cells could maintain its normal electrical activities through spiking activity. This is confirmed in Figs. 10 and 11. An optimal temperature of ion channels could enhance signal transmission during the physiological activities of the cells.

Normal electrical activities of neurons and myocardial cells are very vital in maintaining the normal physiological activities of the nervous and cardiac systems. Injury of these vital systems have been proven fatal and compromising to health. Investigating the cellular changes in electrical activities of cells could be a fruitful avenue in discerning organ physiological activities as well as possible pathological states. Severe sickness and sudden heart disorder are often associated to thermal effect in biological cells^39^. Oakley et al.^40^ reported increase in the number of recorded epileptic seizures in hot weather condition. Understanding the basis of such manifestation is crucial in cardiology, biophysics and several related medical practices. Several contributions have so far enlightened the relationship between cardiac pacing and the initiation of cardiac arrhythmias. Fenton et al.^7^ through numerical experiments reported that such mechanism is closely linked to thermal effect. Lu et al.^41^ observed numerically in a network of neurons, the existence of Arnold tongue-like structure in the time series of membrane potential dependent on temperature parameter. In this current contribution, we have presented a methodology based on analytical and numerical simulation to account for the physiological changes occurring at the cellular level due to temperature fluctuation. Results from our analytical calculations predict thermal couplings promote the growth rate of modulational instability (MI) of the propagating plane wave in the cell lattice. This prediction is confirmed by numerical experiments whereby parameters picked from the unstable region of MI generates a localized wave pattern in the lattice, dependent on the thermal couplings. High thermoelectrical couplings under fixed stimulation current, promotes wave localization. The increase the amplitude of action potential could be at the basis of turbulent electrical activities, observed during the event of sudden heart disorder. Changes in temperature can therefore fluctuate electrical behavior of cardiac tissue. This result partly confirms the investigations of Lu et al.^41^ and Zhao et al.^42^, obtained in the case of neuronal electrical activity. As perspective, a more realistic model can be envisaged to include the physical law of electromagnetic induction and radiation^4,31,43^, time delay^44,45^, noise^46^, autapses^47^ and the action of chemical synapses^48^. Researchers may further build neural circuits coupled with thermistors, whose conductance is temperature dependent in other to investigate cooperative behaviors such synchronization consensus due to heat capture^49^.

## Conclusion

Modulational instability (MI) has been explored in the frame work of the improved myocardial cell model to investigate the effect of temperature fluctuation on electrical activity. By introducing thermoelectrical couplings in the minimal model, we analyze both analytically and numerically via the technique of MI, its effect on wave localization during intercellular communication. Analytically, thermoelectrical couplings considerably modify instability features as the growth rate of MI is observed to be an increasing function of the thermoelectric couplings. By selecting suitable parameters based on our analytical predictions, localized solitonic wave patterns are obtained from the lattice with some features of synchronization. The localization of wave patterns in the network are observed to be dependent on the thermoelectric couplings. High thermoelectric couplings increases the amplitude of myocardial action potential. Temperature fluctuation modifies the nonlinearity of the media and thus promotes the mechanism of MI, since it is known that due to competitive interaction between nonlinearity and dispersion, a small perturbation on the envelope of an impulse plane wave could induce an exponential growth in amplitude, leading to the breakup of the carrier-wave into a train of localized waves. Finally, some open problems are presented for readers extensive investigation.
